# The Association Between Juvenile Onset of Depression and Emotion Regulation Difficulties

**DOI:** 10.3389/fpsyg.2019.02262

**Published:** 2019-10-18

**Authors:** Endre Visted, Lin Sørensen, Jon Vøllestad, Berge Osnes, Julie Lillebostad Svendsen, Sebastian Jentschke, Per-Einar Binder, Elisabeth Schanche

**Affiliations:** ^1^Department of Clinical Psychology, University of Bergen, Bergen, Norway; ^2^Division of Psychiatry, Haukeland University Hospital, Bergen, Norway; ^3^Department of Biological and Medical Psychology, University of Bergen, Bergen, Norway; ^4^Solli District Psychiatric Center (DPS), Nesttun, Norway; ^5^Bjørgvin District Psychiatric Centre, Haukeland University Hospital, Bergen, Norway; ^6^Department of Psychosocial Science, University of Bergen, Bergen, Norway

**Keywords:** emotion regulation, rumination, emotional clarity, depression, recurrent depression

## Abstract

Juvenile onset of Major Depressive Disorder (MDD) is associated with increased likelihood of recurrent episodes of depression and more detrimental clinical trajectories. The aim of the current study was to investigate the effect of juvenile onset of MDD on emotion regulation as measured by self-report and Heart Rate Variability (HRV). Furthermore, we wanted to assess whether juvenile onset impacted the association between rumination and depressive symptoms. Sixty-four individuals with at least three prior episodes of MDD were recruited and filled out self-report questionnaires measuring rumination and emotion regulation abilities. In addition, electrocardiographic assessments were used to calculate HRV. Based on self-reported age of MDD onset, individuals were divided in two groups: Juvenile onset of MDD (first MDD episode before the age of 18, *n* = 30) and adult onset of MDD (first MDD episode after the age of 18, *n* = 34). Results showed that individuals whose first depressive episode occurred in childhood and adolescence reported more rumination and less emotional clarity compared to individuals who had their first episode of MDD in adulthood. Moreover, the tendency to ruminate was strongly associated with depressive symptoms in the juvenile onset of MDD group, whereas no such association was found in the adult onset group. There was no significant group difference for HRV. The findings are discussed in light of existing literature, in addition to suggesting how our findings may inform clinical practice and future research. We conclude that juvenile onset of MDD may lead to difficulties in emotion regulation and that these difficulties may increase depressive symptoms and vulnerability for relapse in this particular subgroup.

## Introduction

The onset of Major Depressive Disorder (MDD) before adulthood is common and has substantial impact on adult functioning and well-being (Lewinsohn et al., 1993, 1999; Patton et al., 2014). Longitudinal studies suggest that younger age of onset of MDD increases the risk of recurrence (Benazzi, 2009; Hardeveld et al., 2012; Hoertel et al., 2017). Compared to individuals with adult onset of MDD, individuals who report early onset of MDD experience more persistent and severe episodes with more complicated clinical features (Zisook et al., 2007; Hybels et al., 2016). Juvenile onset of MDD, that is, before the age of 18, may impair cognitive and emotional functions more than later onset, as this is a period where these capacities are still maturing and developing (Burcusa and Iacono, 2007). Depression early in life could thus disrupt the attainment of developmental milestones with regard to emotion regulation, and could be a factor that can explain the risk of recurrence and increased severity of future depressive episodes (Miller, 2007). It is also possible that emotion regulation difficulties precede depressive symptomatology and may institute a preexisting vulnerability that could lead to the juvenile onset of MDD (McLaughlin et al., 2011). To our knowledge, no prior studies have assessed emotion regulation difficulties in adult participants with a history of MDD in childhood and adolescence. The main aim of the present study was therefore to investigate whether juvenile onset MDD is associated with difficulties in emotion regulation. Furthermore, we wanted to examine whether age of onset impacted the association between maladaptive emotion regulation and depressive symptoms. We used both self-report questionnaires and a proposed psychophysiological marker of emotion regulation ability, Heart Rate Variability (HRV) (Kemp et al., 2010).

### Operationalizing and Measuring Emotion Regulation

Emotion regulation refers to processes that influence which emotions one has, when one has them, and how one experiences or expresses these emotions (Gross, 1998). These processes are often conceptualized as specific strategies used to manage emotional experience, behavior, and physiology (Webb et al., 2012). Research on specific emotion regulation strategies indicates that strategies differ in terms of effectivity and the cognitive and physiological demands they put on the person. Specific strategies, such as rumination, have different consequences on mental health evident by the differential associations with psychopathology in general (Aldao et al., 2010) and with depression in particular (Visted et al., 2018).

People who are prone to depression have a clear tendency to use rumination as an emotion regulation strategy (Joormann and Stanton, 2016). Rumination is defined as the tendency to be repetitively and passively focusing on negative feelings and on the possible causes and consequences of these feelings (Nolen-Hoeksema et al., 2008). Meta-analyses show that rumination is positively associated with depressive symptoms (Aldao et al., 2010; Olatunji et al., 2013). Furthermore, people with current and a history of previous MDD report more rumination compared to healthy controls (Joormann and Stanton, 2016; Liu and Thompson, 2017; Visted et al., 2018). Rumination also has consequences for the course of depression, as initial rumination predicts recurrence of depression (Spasojević and Alloy, 2001) and is associated with prolonged depressed episodes (Robinson and Alloy, 2003). Research on underlying mechanisms for increased rumination in depression suggests that specific cognitive processes may pave the way for increased rumination. For example, previously and currently depressed individuals show limited cognitive control and negative cognitive bias, which both are associated with high levels of rumination (LeMoult and Gotlib, 2018). In addition, those with previous depressive episodes become increasingly vulnerable to ordinary occurrences of negative emotions or thinking, in that these happen more readily and are more difficult to disengage from (Segal et al., 2006). Rumination is thought to activate other elements in a “depressive configuration”, and to reinstate a network of negative thoughts, feelings, and depressive symptoms (Teasdale, 1988). The association between rumination and depression is thought to be strengthened by repeated occurrences of depressive episodes, leading negative repetitive thinking to progressively more easily trigger new episodes of MDD. In sum, habitually employing rumination as a way of regulating negative mood may come with the price of more severe depressive symptomatology as well as prolonged depressed mood and more frequent recurrence of depressive episodes.

It has been argued that the view of emotion regulation as having primarily to do with discrete strategies to modulate emotional experience is too narrow (Gratz et al., 2015). General emotion regulation abilities are suggested as complementary processes that likely influence the selection and successful and context-appropriate implementation of emotion regulation strategies (Tull and Aldao, 2015; Visted et al., 2018). Gratz and Roemer (2004) propose such a framework for general emotion regulation abilities consisting of emotional awareness and clarity and the capacity to tolerate emotions. To apply a specific emotion regulation strategy, the person needs to be aware of the emotion and to be able to understand what the emotion communicates (Sheppes et al., 2015). In addition, being easily overwhelmed by emotions may hinder the use of effective and adaptive emotion regulation strategies and lead to a more rigid use of maladaptive emotion regulation strategies like rumination (Tull and Aldao, 2015).

Empirical evidence supports that increased general emotion regulation abilities have positive impact of effective emotion regulation and mental health. For example, an inability to tolerate negative emotions may increase avoidance and possibly selection of maladaptive emotion regulation strategies (McHugh et al., 2013). Likewise, skillfully identifying and understanding emotions (i.e., emotional clarity) is negatively associated with rumination (Vine et al., 2014). Deficits in general emotion regulation abilities have been linked to depressive symptomatology by way of negative associations between depressive symptoms and emotional clarity (Vine and Aldao, 2014) and tolerance (Preece et al., 2018). Finally, a recent meta-analysis showed that adults with current MDD report limited general emotion regulation abilities, a tendency that seems to persist even after remission of MDD (Visted et al., 2018). Therefore, it seems that general emotion regulation abilities may aid the adaptive selection and implementation of emotion regulation strategies, which in turn may have consequences for mental health. Both emotion regulation strategies and abilities are typically measured by self-report questionnaires, which reflect participant retrospective self-evaluation. Self-report questionnaires are therefore prone to several biases, such as social desirability and recall bias (Althubaiti, 2016). Owing to these methodological limitations, it is required to assess emotion regulation with a broader array of measurement methods. Based on the fact that emotions represent a multifaceted and embodied phenomenon that is dependent on both the central and peripheral nervous system, psychophysiological measures are increasingly incorporated into emotion regulation research.

Evidence suggests that habitual maladaptive emotion regulation and general emotion regulation abilities are associated with physiological processes. This has led investigators to suggest vagus-mediated HRV (vmHRV) to be a peripheral marker of emotion regulation capacity (Appelhans and Luecken, 2006). According to the Neurovisceral Integration Theory, areas in the central nervous system (i.e., frontal cortex areas) that are involved in emotion regulation are also involved in the regulation of the time intervals between successive heartbeats (Thayer and Lane, 2000, 2009; Smith et al., 2017). Prefrontal areas of the cortex have an inhibitory influence on subcortical structures, which subsequently affect vagus nerve input of the heart (Benarroch, 1993). Under rest, the parasympathetic nervous system slows down the heart by the activation of the vagus nerve. Conversely, under challenge or threat, the vagus nerve is deactivated (vagal withdrawal), and the sympathic nervous system is activated to increase heart rate to meet environmental demands (Kemp and Quintana, 2013). Under resting condition, higher vmHRV is thought to indicate flexible vagal regulation of heart rate, and lower resting vmHRV indicates predominant sympathic nervous system activity. A characteristic of populations diagnosed with current MDD is lowered vmHRV in adults (Kemp et al., 2010), adolescents, and children (Koenig et al., 2016), compared to healthy controls. This relative lowering of vmHRV seems to persist after remission of MDD (Bassett et al., 2016), indicating a lack of flexibility of the autonomic nervous system in currently depressed and at-risk populations.

Although recent systematic reviews and meta-syntheses show mixed results (Balzarotti et al., 2017; Holzman and Bridgett, 2017), there is evidence suggesting that self-reported emotion regulation processes coincide with individual differences in vmHRV. For example, lowered vmHRV has shown to be associated with higher use of self-reported rumination (Ottaviani et al., 2016; Williams et al., 2017; Carnevali et al., 2018). Thus, rumination may represent an actual stressor that initiates peripheral physiology (i.e., vagal withdrawal) to resolve the challenge. People who have low vmHRV may therefore lack the ability to inhibit ruminative response styles. There is also evidence that lower vmHRV is associated with limited emotion regulation abilities. For example, a prospective study with adolescents showed that lower vmHRV predicted self-reported limited emotion regulation abilities over 3 years (Vasilev et al., 2009). Further correlational studies show that self-reported ability to describe and identify emotions is positively associated with vmHRV (Williams et al., 2015; Verkuil et al., 2016; Lischke et al., 2018). These results are also supported in performance-based tests that show that people with higher vmHRV better differentiate between emotions, suggesting better emotional clarity and understanding compared to people with low vmHRV (Ruiz-Padial et al., 2003; Park et al., 2012). Thus, greater differentiation and complexity of emotional experience may reflect more flexible and adaptive peripheral physiology (Smith et al., 2017).

### Emotion Regulation and Depression in Childhood and Adolescence

Juvenile-onset depression may impact emotional processes in ways that may lead to difficulties in emotion regulation in adulthood. Childhood and adolescence are important periods where emotion regulation strategies and abilities are acquired through both environmental and biological mechanisms of influence (Zeman et al., 2006). For example, close attachment figures influence emotion regulation development through their provided emotional climate and modeling of emotional practices (Morris et al., 2017). Moreover, maturation of central and peripheral nervous systems enhances emotion regulation flexibility and adaptation (Zeman et al., 2006). Onset of depression in childhood and adolescence is associated with negative cognitive biases and tendencies to ruminate that persist after remission. For example, one prospective study found that children who had experienced a depressive episode reported more pessimistic explanatory styles after remission compared to before the depressive episode (Nolen-Hoeksema et al., 1992). In addition, cross-sectional studies show that previously depressed children report more rumination compared to never-depressed peers (Gibb et al., 2012) and exhibit negative cognitive biases that are associated with rumination (Harrison and Gibb, 2015). Moreover, self-reported emotion regulation abilities like emotional awareness and clarity are negatively associated with depressive symptoms in children and adolescents (Flynn and Rudolph, 2010, 2014; Kranzler et al., 2016; Sendzik et al., 2017). Although juvenile onset of MDD may affect emotional and cognitive functions, initial difficulties with emotion regulation may also lead to early onset of MDD. Prospective studies show that increased rumination predicts later depressive symptoms in children (Abela et al., 2002) and adolescent populations (Broderick and Korteland, 2004; Burwell and Shirk, 2007; Bastin et al., 2015) and predict onset of depressive episodes in children (Gibb et al., 2012) and adolescence (Nolen-Hoeksema et al., 2007). Taken together, both juvenile onset of MDD and preexisting emotion regulation difficulties may increase the likelihood of having difficulties in emotion regulation in adulthood and consequently increasing the risk of recurrent depressive episodes in adulthood. This will also include the likelihood of rumination to be associated with depression through establishing a depressive configuration that is readily reinstated.

### The Present Study

Taken together, separate literatures indicate that juvenile onset of MDD and emotion regulation difficulties are associated with relapse and severity of depressive episodes. In the present study, we defined juvenile onset as experiencing an episode of MDD before the age of 18. Both rumination and general emotion regulation abilities seem to be central factors that may be implicated in the onset and maintenance of depression in the aftermath of juvenile onset of MDD. This includes the strengthened association between rumination and depressive symptoms. Consequently, it is of relevance to investigate these associations with both self-report and psychophysiological measures. To our knowledge, no prior studies have explored self-reported and peripheral proxies of emotion regulation in samples with high risk of recurrence of MDD while simultaneously assessing and taking into account the characteristic of having experienced MDD in childhood or adolescence. Furthermore, no prior study has assessed potential moderating effects of juvenile onset on the association between rumination and depression. Therefore, we wanted to test the following hypotheses:

H1: Juvenile onset of MDD is associated with increased rumination.
H2: Juvenile onset of MDD is associated with limited emotion regulation abilities.
H3: Juvenile onset of MDD is associated with lower autonomic flexibility, as measured by HRV.
H4: Juvenile onset of MDD moderates the association between rumination and depressive symptoms.

Based on existing literature, we expected that individuals who experienced onset of MDD in childhood or adolescence would report more emotion regulation difficulties as measured by self-report-questionnaires and psychophysiological proxies (vmHRV). Given that both rumination and early onset of depression are considered risk factors for recurrent depression, we expected that age of onset would moderate the association between rumination and depressive symptoms. Thus, rumination was expected to be particularly detrimental in individuals with childhood or adolescent onset of MDD.

## Materials and Methods

### Participant Characteristics

To participate, the participants needed to fulfill the following inclusion criteria: (1) 18 years or older, (2) at least three former episodes of MDD, and (3) full or partial remission from depression. Participants were excluded from participation if they (1) fulfilled criteria of a comorbid severe mental disorder (present or life time history of psychosis, schizophrenia, or bipolar disorder); (2) fulfilled criteria for another mental disorder needing treatment, including severe obsessive compulsive disorder, posttraumatic stress disorder, severe eating disorder or borderline personality disorder; (3) fulfilled criteria for current substance use disorders; (4) had any neurological or hormonal diseases; (4) had any prior or current serious cardiovascular disease; (5) attended psychotherapy two or more times per month; (6) had participated in a mindfulness-based intervention in the past 2 years; and (7) were pregnant or lactating.

About half (*n* = 30) of the total sample (*N* = 64) reported to have experienced MDD before the age of 18. The majority of the total sample were white (97%). Regarding education, the majority had completed higher education (university or college; 76.6%), and 23.4% had completed high school. Sixty-one percent were in a full- or part-time job, 17.2% were full-time students in universities or colleges, 12.5% were on sick leave or on social security, and 6.3% were unemployed.

### Sampling Procedures

**FIGURE 1:**
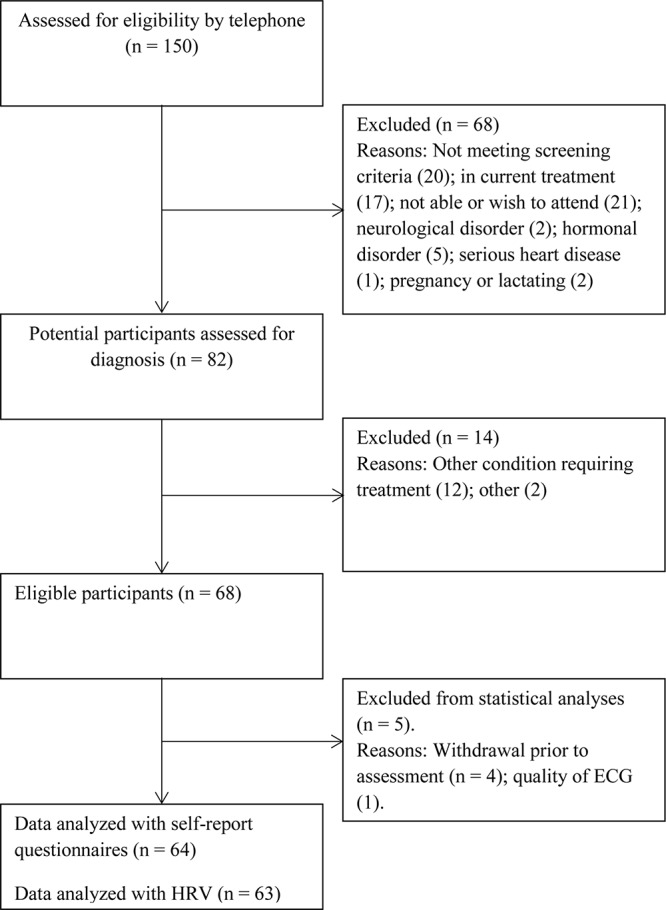
Participant flow.

Participant flow is illustrated in Figure 1. The current study was conducted as a part of a clinical trial investigating the effects of the depression relapse prevention program mindfulness-based cognitive therapy. Therefore, to be able to assess the direct effect of the prevention program, participants that had ongoing psychotherapy for two or more times per month were excluded. In order to ensure that the participants did have an established pattern of recurrent depression, they were required to have experienced at least three prior episodes of depression. The project was preregistered at the ISRCT registry (Trial no. ISRCTN18001392). The current paper reports on the data that were acquired before the prevention program was offered. Participants did not receive any economic compensation but received the prevention program free of charge. The recruitment period was from May 2016 to August 2017. The main route for recruitment was through advertisements posted at offices and waiting rooms of regular general practitioners within the city of Bergen, Norway. In addition, information about the project was posted on mental-health-related forums on social media (Facebook). The advertisement referred to a web page that contained additional information about the project, including a list of exclusion criteria and contact information.

After a preliminary telephone screening, 82 potential eligible participants were interviewed and assessed for eligibility. Diagnostic criteria were assessed using the structured interview MINI International Neuropsychiatric Interview (MINI; Sheehan et al., 1998). The interview assesses Diagnostic and Statistical Manual of Mental Disorders, 4th edition Axis I diagnoses. It covers 17 modules including mood and anxiety disorders, psychotic disorders, eating disorders, alcohol, and substance abuse disorders, and antisocial personality disorder. In addition, participants were screened for borderline personality disorder using the borderline subscale of the Structured Clinical Interview for Diagnostic and Statistical Manual of Mental Disorders, 4th edition (SCID-II; Gibbon et al., 1997). Clinical assessments were conducted by EV and JS, who had undergone comprehensive training in the use of MINI, and had extensive prior experience with using the interview. During the clinical interview, the onset age of depression was assessed by retrospective recall. Accurate estimates were recorded using personal events (e.g., major life events, starting primary/secondary school) as anchor points on a personal time line (Knäuper et al., 1999). In addition, we registered demographic variables, number of previous episodes of MDD, and time in remission. Between 4 and 8 weeks after the clinical interview, the participants met in the laboratory and filled out self-report questionnaires and took part in an electrocardiography (ECG) measurement that was used to calculate resting HRV. To collect ECG data, participants were followed into a soundproof and quite room. After the electrodes were attached, the participants were asked to lie down on a soft bench and asked to relax with their eyes open. Within the research protocol, participants were to fill out a range of questionnaires, in addition to performing two computer tasks and cognitive tests. The total time spent in the laboratory for each participant was ∼3 h. The ethics committee of the Regional Ethics Committee (South East), Norway, approved the study (Reference: 2016/388). Before participation, written consent was obtained, and the study was conducted according to the guidelines of the Declaration of Helsinki.

### Measures and Covariates

#### Difficulties in Emotion Regulation Scale

Emotion regulation abilities were assessed using the Difficulties in Emotion Regulation Scale (DERS) (Gratz and Roemer, 2004). DERS is a 36-item questionnaire that measures six facets of difficulties in emotion regulation [Cronbach’s alpha (α) from the present study are presented within the parentheses]: (1) difficulties with accepting negative emotions (DERS NON-ACCEPT, α = 0.90), (2) difficulties engaging in goal-directed behavior (DERS GOALS, α = 0.83), (3) impulse control difficulties (DERS IMPULSE, α = 0.74), (4) limited access to emotion regulation strategies (DERS STRATEGIES, α = 0.84), (5) lack of emotional awareness (DERS AWARENESS α = 0.75), and (6) lack of emotional clarity (DERS CLARITY, α = 0.83). In addition, a total score is obtained by adding all scales into one score (DERS TOTAL, α = 0.92). The items are rated on a 5-point scale ranging from 1 [almost never (0–10%)] to 5 [almost always (91–100%)]. Higher scores on each subscale indicate more difficulties within each facet.

#### Ruminative Response Scale

Rumination was assessed by the 10-item version of ruminative response scale (RRS) (Treynor et al., 2003). This version consists of two subscales that measure styles of responding when experiencing negative emotions on a scale from 1 (almost never) to 4 (almost always). Whereas RRS REFLECTION measures a voluntary focus of attention to engage in cognitive problem solving to alleviate one’s depressive symptoms, the RRS BROODING assesses a passive comparison of one’s current situation with some unachieved standard. The short 10-item version of the RRS was used in the present study because the original 24-item version contained a depressive rumination scale that had too much overlap with depressive symptoms (Treynor et al., 2003). Moreover, the 10-item RRS recently showed good scale reliability and validity for samples with current MDD (Parola et al., 2017). Cronbach’s alphas were acceptable for RRS TOTAL (α = 0.77), RRS REFLECTION (α = 0.74), and RRS BROODING (α = 0.67).

#### Beck Depression Inventory II

To assess depressive symptoms over the past 2 weeks, the Beck Depression Inventory II (BDI-II) (Beck et al., 1996) was used. BDI-II consists of 21 items that assesses a range of emotional, somatic, and cognitive symptoms of depression. The Cronbach’s alpha from the current investigation was acceptable (α = 0.88).

#### Electrocardiography

Electrocardiography (ECG) signals were obtained using a standard 12-lead resting ECG in supine position, following established standard guidelines (Task Force of the European Society of Cardiology the North American Society of Pacing Electrophysiology, 1996). The ECG was recorded over a period of ∼5 min at 1,000 Hz sampling rate using disposable electrodes connected to a made-to-order ECG device (Research and Transfer Center at the University of Applied Sciences Leipzig, Germany). This device transmitted data via a Bluetooth connection, thereby ensuring both patient safety (being battery powered) and signal quality (line noise in the recording was greatly reduced by not having a cable connection to the recording PC).

#### Heart Rate Variability

The calculation of resting HRV was carried out using Kubios (Tarvainen et al., 2014). Two measures served as indicators of resting vmHRV: (1) the square root of the mean squared differences of successive RR interval values (RMSSD), (2) the high-frequency (HF-HRV; 0.15–0.4 Hz) component of the respiratory sinus arrhythmia. A more detailed description is given under Supplementary Material.

### Statistical Procedures

All statistical tests were conducted in IBM SPSS version 25. In preparation for the statistical analyses, variables were recoded and inspected for outliers and normal distribution, HRV measures were transformed (e.g., when not normally distributed), and missing values were replaced: (1) the age of onset variable was dichotomized (0 = age of onset ≥ 18 years old; 1 = age of onset ≤ 17 years old). (2) self-report variables were inspected for outliers, and skewness and kurtosis were inspected to ensure normal distribution. Shapiro–Wilks tests conducted on HRV variables indicated that these variables were not normally distributed (RMSSD and HF-HRV; *p* < 0.05). The raw HRV scores were therefore log-transformed to approximate normal distributions (LN RMSSD, LN HF-HRV). (3) The dataset had a limited number of missing items, within the scales DERS NON-ACCEPT (two missing responses), DERS IMPULSE (one missing response), DERS AWARENESS (five missing responses), DERS CLARITY (one missing response), BDI-II (nine missing responses), and RRS BROODING (one missing response). For all variables containing missing values, comparison of means and covariances were conducted using Littles’ missing completely at random test (Little, 1988). The test indicated that missing data were randomly distributed (all *p*s > 0.24). Missing values were therefore replaced using the expectation maximization algorithm in SPSS.

For descriptive summaries of our samples, means and standard deviations were calculated. In addition, we conducted the following inference statistics: (1) *t*-tests and chi-square tests were conducted with the dichotomized age of onset as grouping variable to assess differences between groups on background variables (demographic and clinical variables). (2) Bivariate correlations were calculated to assess association between emotion regulation variables and depressive symptoms. (3) Group differences based on age of onset were calculated by conducting univariate analyses of covariance (ANCOVA), with dichotomized age of onset as fixed factor, and clinical variables including DERS subscales and RRS subscales as dependent variables and age and depressive symptoms as covariates. We planned to use age as a covariate because we, based on prior studies, expected younger age in early age of onset group (e.g., Weinberg et al., 2016; Clark et al., 2018). We controlled for current depressive symptoms to assess whether early onset of MDD predicts emotion regulation difficulties over and above concurrent symptom levels. We expected high correlations between emotion regulation variables and current depressive symptoms. Therefore, to avoid problems with multicollinearity when controlling for depressive symptoms, the variance in depressive symptoms that was explained by emotion regulation variables was extracted into residual score variables (i.e., residual scores of depressive symptoms: the variance explained by the rumination and DERS scores was extracted in linear regression analyses run before the main ANCOVA analyses). To control for confounding variables of HRV, we additionally added gender, body mass index, nicotine consumption (cigarettes per week), and medication (antidepressant, blood thinner, and blood pressure) as covariates. False discovery rate correction was applied to control for multiple comparisons (Benjamini and Hochberg, 1995). All *p*-values reported are non-corrected because *p* < 0.05 were significant after false discovery rate correction. (4) Effect sizes for each comparison were calculated using the partial eta squared and interpreted in line with established guidelines (0.01 is considered a small effect size; 0.06 a medium effect size; 0.14 a large effect size; Cohen, 1988). (5) Moderation effects of age of onset on the associations between rumination and depressive symptoms were assessed using the PROCESS macro for IBM SPSS 25 (Hayes, 2018). In our moderation analysis, RRS BROODING was used as the independent variable, BDI-II total score as dependent variable, and age of onset (dichotomized variable) as moderator. We chose RRS BROODING as an indicator for rumination because this subscale reflects a passive comparison of one’s current situation with some unachieved standard, which is a key feature of rumination. The regression analysis was computed with centered means and 95% bootstrapping of 5,000 resamples. The heteroscedasticity-consistent standard errors estimator was used (HC3) was used to minimize the effect of heteroscedasticity on statistical inferences from our analyses (Hayes and Cai, 2007). Interaction term and statistical tests of simple slopes are automatically computed using the PROCESS macro. The form of the interaction was inspected graphically and statistically, by examining the 95% bootstrapped confidence intervals for the effect of age of onset on BDI-II at each level of the moderator.

## Results

### Participant Characteristics and Correlation Analyses

Participant characteristics are presented in Table 1. The early onset MDD group was significantly younger (*M* = 34.60, *SD* = 11.96) than the late-onset MDD group [*M* = 44.88, *SD* = 11.66, *t*(62) = −3.48, *p* = 0.001]. Participants in the juvenile onset group had lower onset age (*M* = 11.80, *SD* = 3.16, range = 6–17) compared to the adult onset group [*M* = 27.59, *SD* = 8.78; range = 18–46; *t*(62) = -9.33, *p* < 0.001]. Moreover, a higher number of participants with early onset of MDD had experienced 10 or more previous episodes of MDD compared to participants with adult onset. No other demographic or clinical variables was significantly different between the two groups, including months in remission [*t*(61) = −1.25, *p* = 0.217] and current depressive symptoms [*t*(62) = 0.75, *p* = 0.457].

**TABLE 1:** Demographic, clinical, and background variables.

	**Total**	**Juvenile**	**Adult**	
	**sample**	**onset**	**onset**	
***N***	**64**	**30**	**34**	***p***
**Demographic variables**				
% Female	73.4	76.70	70.60	0.583
Age (*M*, *SD*)	40.06 (12.80)	34.60 (11.96)	44.88 (11.66)	0.001
BMI (*M*, *SD*)	25.81 (4.82)	25.00 (5.42)	26.51 (4.20)	0.217
**Antidepressant (*n*)**				
SSRI	13	7	6	0.573
Tetracycline	2	1	1	0.928
Aminoketon	3	2	1	0.482
**Clinical variables**				
Age of onset (*M*, *SD*)	20.19 (10.39)	11.80 (3.16)	27.59 (8.78)	>0.001
Months in remission	16.68 (43.93)	9.24 (16.63)	23.03 (57.45)	0.217
(*M*, *SD*)				
≥10 previous episodes	22	15	7	0.013
of MDD (*n*)				
BDI-II (*M*, *SD*)	12.80 (8.09)	13.61 (9.27)	12.09 (6.95)	0.457
**Comorbidity (*n*)**				
Panic disorder current	3	3	0	0.059
Panic disorder prior	13	5	8	0.496
Agoraphobia	9	6	3	0.199
Social anxiety	3	3	0	0.059
GAD	12	7	5	0.688
Any anxiety disorder	29	14	15	0.479

*BMI, Body Mass Index; SSRI, Selective Serotonin Reuptake Inhibitors; BDI-II, Becks Depression Inventory II; GAD, Generalized Anxiety Disorder.*

All correlations between variables are presented in Table 2. The majority of DERS subscales correlated in predicted directions with maladaptive rumination and depressive symptoms. Neither DERS scores nor rumination correlated with either vmHRV components. Log-transformed RMSSD correlated significantly with depressive symptoms (*r* = −0.25, *p* < 0.05). However, this correlation did not survive when controlling for covariates of age, body mass index, gender, and medication.

**TABLE 2:** Zero-order correlations for all participants.

	**1.**	**2.**	**3.**	**4.**	**5.**	**6.**	**7.**	**8.**	**9.**	**10.**	**11.**	**12.**
1. BDI-II	–											
2. RRS TOTAL	0.215	–										
3. RRS BROODING	0.351^∗^	0.825^∗∗^	–									
4. RRS REFLECTION	0.002	0.845^∗∗^	0.395^∗∗^	–								
5. DERS TOTAL	0.514^∗∗^	0.433^∗∗^	0.523^∗∗^	0.208	–							
6. DERS NON-ACCEPT	0.309^∗^	0.517^∗∗^	0.554^∗∗^	0.317^∗^	0.793^∗∗^	–						
7. DERS GOALS	0.382^∗^	0.303^∗^	0.283^∗^	0.224	0.746^∗∗^	0.520^∗∗^	–					
8. DERS IMPULSE	0.319^∗^	0.306^∗^	0.361^∗^	0.155	0.732^∗∗^	0.443^∗∗^	0.710^∗∗^	–				
9. DERS AWARENESS	0.194	–0.128	0.042	−0.249^∗^	0.322^∗^	0.144	–0.066	–0.103	–			
10. DERS STRATEGIES	0.481^∗∗^	0.408^∗∗^	0.474^∗∗^	0.214	0.877^∗∗^	0.643^∗∗^	0.636^∗∗^	0.676^∗∗^	0.101	–		
11. DERS CLARITY	0.411^∗∗^	0.258^∗^	0.351^∗^	0.087	0.568^∗∗^	0.315^∗^	0.189	0.251^∗^	0.498^∗∗^	0.320	–	
12. LN RMSSD^a^	−0.250^∗^	–0.065	–0.061	–0.048	–0.009	0.011	–0.058	–0.044	–0.051	–0.042	0.070	–
13. LN HF-HRV^a^	–0.202	–0.010	–0.002	–0.015	0.020	0.094	–0.044	0.005	–0.010	0.012	0.024	0.964^∗∗^

*BDI-II, Becks Depression Inventory II; RRS, Ruminative Responses Scale; DERS, Difficulties in Emotion Regulation Scale; LN RMSSD, log-transformed Root Mean Square of Successive Differences; LN HF-HRV, log-transformed High Frequency Heart Rate Variability. ^*a*^*N* = 63. ^∗^*p* < 0.05; ^∗∗^*p* < 0.001.*

### Between Group Analyses

Analyses of covariances (Table 3) revealed significant differences in DERS CLARITY scores and RRS BROODING, when controlling for age and current depressive symptoms. None of the vmHRV components were significantly different. Effect sizes yielded medium effects of early age of onset on rumination (RRS BROODING) and emotional clarity (DERS CLARITY).

**TABLE 3:** Means, standard deviations, and between-group analyses (analyses of covariance) for emotion regulation variables, controlled for age and depressive symptoms.

	***M* (*SD*)**			**ANCOVA**		**ES**
	**Total sample**	**Juvenile onset**	**Adult onset**	***F***	***p***	**η^2^_p_**
RRS BROODING	13.52 (3.08)	14.60 (2.86)	12.57 (2.99)	4.30	0.042	0.067
RRS REFLECTION	12.72 (3.25)	13.30 (2.74)	12.21 (3.60)	0.64	0.427	0.011
DERS NON-ACCEPT	18.12 (6.08)	18.83 (5.79)	17.49 (6.34)	0.17	0.682	0.003
DERS GOALS	17.56 (4.43)	17.63 (4.70)	17.50 (4.24)	0.25	0.620	0.004
DERS IMPULSE	14.92 (4.41)	15.63 (5.10)	14.29 (3.65)	0.98	0.326	0.016
DERS AWARENESS	16.29 (3.93)	16.05 (4.41)	16.50 (3.50)	0.36	0.554	0.006
DERS STRATEGIES	22.75 (6.37)	23.13 (6.95)	22.41 (5.89)	0.35	0.555	0.006
DERS CLARITY	12.51 (4.05)	13.90 (4.23)	11.29 (3.51)	4.15	0.046	0.065
LN RMSSD	3.40 (0.71)	3.50 (0.72)	3.31 (0.70)	0.43	0.512	0.002
LN HF-HRV	5.86 (1.57)	6.10 (1.58)	5.67 (1.59)	0.45	0.505	0.003

*RRS, Ruminative Responses Scale; DERS, Difficulties in Emotion Regulation Scale; LN RMSSD, log-transformed Root Mean Square of Successive Differences; LN HF-HRV, log-transformed High Frequency Heart Rate Variability.*

### Moderation Analysis

The relationship between rumination and depressive symptoms was not significant [*b* = 1.08, *SE* = 0.64, *t* = 1.69, *p* = 0.096, 95% CI (−0.20, 2.36)]. However, there was a statistical difference between the groups in that the relationship between rumination and depressive symptoms was not significant with adult onset depression [*b* = 0.51, *SE* = 0.41, *t* = 1.25, *p* = 0.215, 95% CI (−0.31, 1.33)] but significant with participants that experienced juvenile onset of MDD [*b* = 1.59, *SE* = 0.49, *t* = 3.27, *p* = 0.002, 95% CI (0.62, 2.57)].

## Discussion

The main aim of the present study was to explore self-reported and peripheral proxies of emotion regulation in samples with high risk of recurrence of MDD, while also assessing and considering the characteristic of having experienced MDD in childhood or adolescence. Results showed that the onset of MDD before the age of 18 was associated with increased rumination and decreased emotional clarity. In addition, juvenile onset of depression strengthened the association between rumination and depressive symptoms, suggesting that rumination may constitute an increased risk of recurrence of depression in this particular subgroup. However, no significant differences were found for the psychophysiological correlate of emotion regulation, the vmHRV.

Earlier studies have shown that increased rumination predicts recurrent depressive episodes (Spasojević and Alloy, 2001) and longer duration of these episodes (Robinson and Alloy, 2003). Our findings suggest that juvenile onset of MDD may increase the likelihood of habitual rumination and may contribute to recurrent episodes. The finding is also in line with longitudinal findings that children who become depressed may incorporate negative cognitions that persist after the depressive symptoms remit (Nolen-Hoeksema et al., 1992) and that previously depressed children report more rumination compared to never-depressed peers (Gibb et al., 2012). The tendency to increased rumination in depression has been linked to underlying negative cognitive biases and limited cognitive control, which pave the way for maladaptive emotion regulation strategies (LeMoult and Gotlib, 2018). Evidence indicates that depression in younger age is associated with negative cognitive bias. For example, children with current MDD avoided looking at negative stimuli to a greater degree compared to healthy children (Harrison and Gibb, 2015). Moreover, ambiguous scenarios were interpreted as more negative and less pleasant by depressed adolescents when compared to never-depressed peers (Orchard et al., 2016) as well as by adolescents in risk of developing MDD (offspring of depressed mothers; Dearing and Gotlib, 2009). The existing literature thus indicates that depression in childhood and adolescent distorts cognitive development and consequently interferes with adaptive emotion regulation. Although the present study is cross-sectional and cannot lead to firm conclusions about directionality, our findings are nevertheless supportive of the vulnerability found in individuals with juvenile onset of depression from other studies.

In addition, our moderation analysis indicated a strengthened association between rumination and depressive symptoms in individuals that had onset of depression in childhood and adolescence. This indicates that rumination may be particularly disruptive for individuals with a juvenile onset of MDD. In this group, habitual rumination may more readily activate elements in a “depressive configuration” and thereby potentially contribute to a full-blown depressive episode.

We also found that juvenile onset depression was associated with lower emotional clarity. Individuals who experienced depression before the age of 18 report more difficulties in understanding and differentiating their emotional reactions compared to people who experienced onset of depression in adulthood. Difficulties with identifying and understanding emotions render selection and implementation of context-appropriate emotion regulation strategies difficult (Sheppes et al., 2015). This may also explain the increased use of rumination as an emotion regulation strategy. Vice versa, rumination may hinder the development of adaptive understanding of their emotional experiences and might therefore be a risk factor for poor development of emotional clarity (Haas et al., 2018). Rumination may therefore be both a way of regulating emotions that are poorly understood and differentiated, as well an obstacle to the development of emotional clarity.

Although our findings suggest that juvenile onset of MDD may lead to difficulties in rumination and emotional clarity in adulthood, we did not find any differences in the other facets of emotion regulation difficulties. However, it must be emphasized that the DERS scores of both subgroups indicate substantial difficulties in emotion regulation across all facets. They are thus well into the clinical domain with regard to these difficulties (Neacsiu et al., 2017). Therefore, detecting statistically significant differences between the subgroups where all the individuals have recurring depression is less likely. The fact that differences in rumination and emotional clarity do emerge with moderate effect sizes is notable and worthy of further investigation.

Finally, no significant associations were found between age of onset of MDD and resting vmHRV, proposed as a psychophysiological correlate of emotion regulation. There are three possible explanations for the failure to observe a significant group difference: First, the majority of previous studies assessing the relation between emotion regulation and resting vmHRV did this either in correlational studies or comparing healthy controls to a clinical group (Kemp et al., 2010; Koenig et al., 2016; Holzman and Bridgett, 2017). Comparing this relation between two clinical subgroups makes the expected effect size relatively small, and lack of significant findings quite probable. The heterogeneity when it comes to clinical features of depression also adds to the difficulty observing a significant difference between these two subgroups (Monroe and Anderson, 2015). Previous evidence suggests that the vagal influence on the heart as measured by resting vmHRV may be relatively subtle, so that very large samples are needed to detect significant effects, rendering the clinical utility of the resting vmHRV limited (Schumann et al., 2017). Second, it could be that the impact of early onset MDD on cognitive and emotional processes is too subtle to be measured by current physiological assessment methods. The evidence for this assumption is mixed: On the one hand, former studies (in adults) observed that lower vmHRV is associated with both increased rumination (Williams et al., 2017; Carnevali et al., 2018) and limited emotional clarity (Ruiz-Padial et al., 2003; Park et al., 2012; Verkuil et al., 2016; Lischke et al., 2018). On the other hand, recent meta-analyses have shown that the association between self-regulatory capacities and vmHRV is relatively weak and inconsistent. For example, a meta-analysis summarizing 52 studies of the relation between emotion regulation capacity and vmHRV yielded a small effect size (*r* = 0.10; Holzman and Bridgett, 2017). Similarly, a recent systematic review of studies of emotion regulation outcomes and HRV in healthy individuals concluded that existing evidence is mixed with no clear picture (Balzarotti et al., 2017). Third, the type of HRV measurement (i.e., resting vmHRV) may have not been the most appropriate measure: According to a recent review, HRV measurement during stress induction (phasic HRV) may yield more information and give a clearer picture of autonomic flexibility than resting HRV in clinical populations (Schiweck et al., 2018). Individuals with MDD typically show blunted reactivity in HRV (i.e., HRV does not decrease during stress), which suggest that their autonomic nervous system does not respond sufficiently to environmental demands. Phasic HRV may therefore yield information about the actual autonomic processes that may be missed during resting HRV measurements. Generally, the relationship between HRV and vagal modulation of the heart is likely to be very complex with large between-subject variation and sensitivity to a range of covariates (Quintana and Heathers, 2014).

The current study has several limitations. Most notably, the study is based on cross-sectional data, which limits the understanding of the directionality of associations. It is still unclear when emotion regulation difficulties emerged. We do not know if individuals with increased rumination and limited emotional clarity are more prone to depression in childhood or whether early depression *per se* is causing these emotion regulation difficulties. Moreover, it is unclear whether more severe depression and greater number of depressive episodes lead to emotion regulation difficulties in adulthood. In addition, we cannot know whether it is the juvenile onset *per se* or the accumulated number of depressive episodes that are responsible for possible associations between rumination and depression. To identify the temporal pattern of emotion regulation difficulties and depression among those with juvenile onset of MDD, longitudinal studies are needed. These studies should include records of both accurate age of onset, and number of depressive episodes, to better understand the exact contributions of each of these factors. However, cross-sectional findings may inform more resource demanding longitudinal designs.

Prospective studies have found that initial heightened rumination predict future depressive symptoms (Abela et al., 2002; Broderick and Korteland, 2004; Burwell and Shirk, 2007; Bastin et al., 2015) and onset of MDD (Nolen-Hoeksema et al., 2007; Gibb et al., 2012) in children and adolescents. Similar findings are reported with general emotion regulation abilities in that limited emotional awareness and clarity was found to predict future depressive symptoms (Kranzler et al., 2016). However, it is possible that preexisting difficulties in emotion regulation in combination with early onset of MDD will increase subsequent recurrence of depressive episodes. For example, adolescents who experienced an episode of depression in addition to having limited adaptive coping skills typically had subsequent recurrence of depression to a greater degree than peers with adaptive coping skills (Hammen et al., 2008). Therefore, adaptive coping skills and emotion regulation may have protected adolescents that did not experience recurrent depressions after early onset. Another prospective study that followed an adolescent population over 12 years showed that both preexisting risk factors (e.g., parental history of depression) and early onset of depression predicted recurrent episodes of depression (Pettit et al., 2013). It is therefore likely that both preexisting vulnerabilities like emotion regulation difficulties and early onset of MDD may increase the likelihood of subsequent recurrent depressive episodes.

A further limitation is related to the use of self-reports of emotion regulation. Self-report measures have methodological caveats and may thus limit validity of our findings (Althubaiti, 2016). Relatedly, the retrospective recall of first episode of MDD may not be accurate. However, we aimed at increasing the accuracy of recall using established guidelines (Knäuper et al., 1999). In addition, operating with a dichotomization of age of onset based on 18 years to define juvenile depression could have some disadvantages. The exact definition of juvenile onset varies in the field of depression research. It could be argued that the particular sensitive period with regard to the onset of depression could be of both shorter or longer duration. In that sense, the age of 18 is somewhat arbitrary. However, the age of 18 is considered as a cultural transitional point to adulthood in western Europe and can be argued to have a certain ecological validity. Furthermore, it is in accordance with clinical research which often draws the line between adolescent and adult populations at the age of 18. Another limitation was the lack of interrater reliability tests for the clinical interviews. Adding such a measure would possibly increase the reliability and validity of the participants’ diagnostic status. Finally, the sample is relatively small and ethnically homogenous, limiting the generalizability of our findings.

Future studies should use prospective designs, investigate ruminative response styles and emotional clarity in childhood and adolescence, and assess risk of onset and recurrence. In addition, using behavioral measures to assess emotional clarity could increase validity of our findings. For example, the use of experience sampling provides ecologically valid assessments of participants emotional clarity and differentiation (Kashdan et al., 2015). Furthermore, laboratory investigations of juvenile onset MDD samples may further increase our knowledge of emotion regulation capacity of this group. For example, instructed or spontaneous emotion regulation after emotional induction may yield differential effects on actual emotion regulation strategy use. Likewise, phasic HRV measurements should be investigated in this population to investigate effects of actual autonomic responding to different environmental demands. The inclusion of such measures may yield a clearer picture of autonomic flexibility.

Our findings may inform both prevention and psychological interventions. First, decreasing rumination and increasing emotional clarity in at-risk populations through psychoeducation may increase resilience against depression. Our study suggest that special focus should be on reducing rumination in patients with early onset depression, as this is associated with increased depressive symptomatology in this subgroup. In both individual and group-based psychotherapeutic interventions, special focus on both rumination and emotional clarity may yield better results and potentially reduce relapse and recurrence. Interestingly, third-generation cognitive therapies like metacognitive therapy (Wells et al., 2009) and mindfulness-based cognitive therapy (Segal et al., 2013) seek to decrease ruminative response styles and increase awareness and understanding of emotions. Finally, teaching patients emotional vocabulary by integrating bodily reactions (i.e., interoceptive information and facial expressions) to emotion words may also decrease relapse and recurrence of depressive episodes.

We conclude that the onset of depression in childhood and adolescence is associated with increased rumination and limited understanding and clarity of emotions. Rumination also seems to add extra risk in individuals who experienced early onset of MDD, as the association between rumination and depressive symptoms is strengthened. This in turn could lead to recurrence of depressive episodes.

## Data Availability Statement

The datasets generated for this study are available on request to the corresponding author.

## Ethics Statement

The studies involving human participants were reviewed and approved by the Regional Ethics Committee (South East), Norway Reference: 2016/388. The patients/participants provided their written informed consent to participate in this study.

## Author Contributions

EV, ES, LS, BO, and P-EB: study design. EV, LS, JV, BO, JS, P-EB, and ES: data collection. EV, LS, JS, and SJ: data preparation and analysis. LS and SJ: supervision statistics and HRV. EV: writing of first draft of manuscript. EV, LS, JV, BO, SJ, P-EB, and ES: revision of manuscript.

## Conflict of Interest

The authors declare that the research was conducted in the absence of any commercial or financial relationships that could be construed as a potential conflict of interest.
