# Supplementary material for: The Association Between Juvenile Onset of Depression and Emotion Regulation Difficulties
Source: Front Psychol. 2019 Oct 18;10:2262. doi: 10.3389/fpsyg.2019.02262 (PMC6816416; doi:10.3389/fpsyg.2019.02262)
Supplement: Supplementary file 1 [file Presentation_1.pdf]

Heart Rate Variability (HRV). The calculation of resting HRV was carried out using Kubios (Tarvainen, Niskanen, Lipponen, Ranta-aho, & Karjalainen, 2014). In preparation for this calculation, the data were converted from raw data (as acquired by the ECG device) into European Data Format (EDF). During import, artifacts were removed using three different methods proposed in Sörnmo & Laguna (2005): [1] Data were decimated (i.e., the sample rate was decreased to 50 Hz in steps of integers – typically 2 or 5). The decimated data were low-pass filtered (with a cut-off frequency of 0.5 Hz) consequently only containing slow-drift-components which were interpolated back to the original sampling rate and subtracted from the original data (thereby removing slow-drift-components). [2] Fitting of a polynomial spline to a point within the isoelectric line (i.e., the period with little heart activity between two PQRST waveforms, the point was set to the middle between two R-peaks) and removing the slow-drift-components caught by the spline by subtracting it from the original data. [3] Estimation-subtraction of frequency components representing line noise (i.e., 50 Hz) by first determining the peak frequency in a window from 49 to 51 Hz, then fitting a sine wave with this peak frequency to the data and removing the fitted data (thereby removing the frequency components representing the line noise while leaving the rest of the frequency spectrum intact). Because of the construction of the ECG device (battery powered with wireless data transmission) and the features of the measurement situation (participants were lying relaxed in supine position in a quiet environment) the two main artifacts – line noise and slow drifts, typically mainly due to movement during ECG acquisition – were already minimized so that the influence of these artifact removal procedures on the data is regarded minimal. After the import the software automatically detected heart beats (at the R-peak, using V3). These automatically detected heartbeats were visually inspected to identify erroneous heartbeats and missing R-peaks. If visual inspection indicated an unsatisfactorily detection, two strategies were followed: [1] Re-detection using an alternative channel, typically V2 or V4). [2] In a

very limited number of cases, R-peaks were manually corrected: In three participants, seven erroneous R-peaks were deleted, and two R-peaks were added because of insufficient data quality caused by movement of the participant. After correction, distances between adjacent R-peaks were derived and used for further analyses. For one participant, the data quality was so insufficient that s/he had to be removed from further analyses. No detrending was used within the HRV analysis. Two measures served as indicators of resting vmHRV: [1] RMSSD, denoted as measure of the short-term variability, was calculated as the square root of the mean squared differences of successive RR interval values. [2] The high frequency (HF-HRV; 0.15 to 0.4 Hz) component of the respiratory sinus arrhythmia (RSA) was calculated as the integral of that frequency range based upon an estimation of the power spectral density (PSD). This estimation used autoregressive (AR) time series modeling, where the time series of successive RR intervals is modeled as an AR(p) process with 16 parameters.

References

Sörnmo, L., & Laguna, P. (2005). *Bioelectrical Signal Processing in Cardiac and Neurological Applications.* Elsevier/Academic Press, Amsterdam.

Tarvainen, M. P., Niskanen, J.-P., Lipponen, J. A., Ranta-aho, P. O., & Karjalainen, P. A. (2014). Kubios HRV – Heart rate variability analysis software. *Computer Methods and Programs in Biomedicine, 113*(1), 210-220. doi:10.1016/j.cmpb.2013.07.024
